# Aroma Release in Wine Using Co-Immobilized Enzyme Aggregates

**DOI:** 10.3390/molecules21111485

**Published:** 2016-11-08

**Authors:** Katherine Ahumada, Ana Martínez-Gil, Yerko Moreno-Simunovic, Andrés Illanes, Lorena Wilson

**Affiliations:** 1 School of Biochemical Engineering, Pontificia Universidad Católica de Valparaíso, Valparaíso 2362803, Chile; elisabet.ahumada@gmail.com (K.A.); andres.illanes@pucv.cl (A.I.); 2 Technological Center for Grapevine and Wine, Faculty of Agricultural Sciences, Universidad de Talca, Talca 824000, Chile; a.martinez@utalca.cl (A.M.-G.); ymoreno@utalca.cl (Y.M.-S.)

**Keywords:** glycosidases, wine, aroma, combi-CLEAs

## Abstract

Aroma is a remarkable factor of quality and consumer preference in wine, representing a distinctive feature of the product. Most aromatic compounds in varietals are in the form of glycosidic precursors, which are constituted by a volatile aglycone moiety linked to a glucose residue by an *O*-glycosidic bond; glucose is often linked to another sugar (arabinose, rhamnose or apiose). The use of soluble β-glycosidases for aroma liberation implies the addition of a precipitating agent to remove it from the product and precludes its reuse after one batch. An attractive option from a technological perspective that will aid in removing such constraints is the use of immobilized glycosidases. Immobilization by aggregation and crosslinking is a simple strategy producing enzyme catalysts of very high specific activity, being an attractive option to conventional immobilization to solid inert supports. The purpose of this work was the evaluation of co-immobilized β-glycosidases crosslinked aggregates produced from the commercial preparation AR2000, which contains the enzymes involved in the release of aromatic terpenes in Muscat wine (α-l-arabinofuranosidase and β-d-glucopyranosidase). To do so, experiments were conducted with co-immobilized crosslinked enzyme aggregates (combi-CLEAs), and with the soluble enzymes, using an experiment without enzyme addition as control. Stability of the enzymes at the conditions of winemaking was assessed and the volatiles composition of wine was determined by SPE-GC-MS. Stability of enzymes in combi-CLEAs was much higher than in soluble form, 80% of the initial activity remaining after 60 days in contact with the wine; at the same conditions, the soluble enzymes had lost 80% of their initial activities after 20 days. Such higher stabilities will allow prolonged use of the enzyme catalyst reducing its impact in the cost of winemaking. Wine treated with combi-CLEAs was the one exhibiting the highest concentration of total terpenes (18% higher than the control) and the highest concentrations of linalool (20% higher), nerol (20% higher) and geraniol (100% higher), which are the most important terpenes in determining Muscat typicity. Co-immobilized enzymes were highly stable at winemaking conditions, so their reutilization is possible and technologically attractive by reducing the impact of enzyme cost on winemaking cost.

## 1. Introduction

Aroma is undoubtedly, one the most remarkable quality factors appreciated by the consumers, providing an identity stamp to wines. Wine aroma is generated after a complex process involving a great number of chemical and enzymatic reactions. It is well known that compounds from grapes play the most important role in the aromatic characteristics of the variety. These compounds are referred to as varietal aroma, which consists on specific precursors, free compounds, and compounds such as amino acids or fatty acids whose profile is characteristic of the variety. Most of the varietal aroma compounds are in their glycosidic form. These compounds are constituted by a volatile aglycone which is linked to a glucose moiety by an *O*-glycosidic bond, being this glucose usually attached to another sugar (arabinose, rhamnose or apiose). If these bonds are not broken down, these compounds will remain undetected by the human nose, highlighting the importance of breaking them during winemaking. This is possible by the action of endogenous or exogenous enzymes with β-glycosidase activity or by acid hydrolysis. All grape varieties have this type of precursors, but the variety having them in highest concentration is Muscat, being the released terpenes those providing their aromatic typicity and their aromatic quality [1] due to their low olfactory detection threshold.

**Scheme 1:**
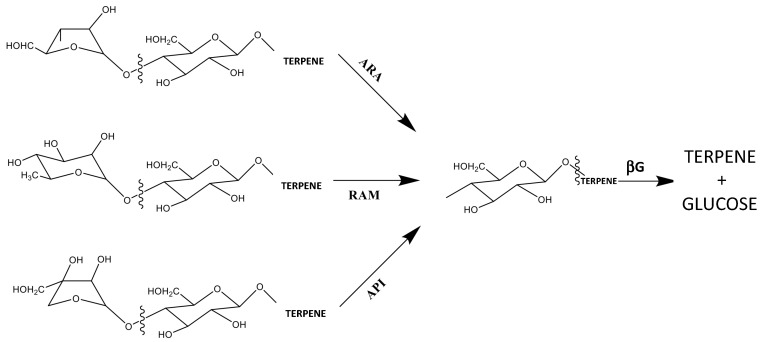
Scheme of reactions involved in aroma release in wine.

As shown in Scheme 1, the enzymatic hydrolysis of monoterpene glycoconjugates is a sequential process of two consecutive reactions: the first one is the dissociation of the monoterpenyl β-d-glycoside from its corresponding residual sugar, which is catalyzed by three glycosidases: α-l-arabinofuranosidase (ARA), α-l-rhamnopyranosidase (RAM) and β-d-apiofuranosidase (API); the second one is the liberation of monoterpenes catalyzed by β-d-glucopyranosidase (βG), also known as β-glucosidase. Once volatile terpenes are released into the medium they make part of the aromatic fraction of wine [2,3].

Enzyme immobilization is a widely used strategy for the application of enzymes in industrial processes due to the advantages of using an insoluble catalyst and the likely increase in enzyme stability under operational conditions. There is now an ample spectrum of methods for enzyme immobilization [4,5]; however, the best one will vary from case to case and many variables will be involved in optimizing the immobilization process. The most used technique for enzyme immobilization consists in the attachment or containment of the enzyme to (into) a solid support (carrier-bound); the resulting catalyst can be easily separated from the reaction medium producing in most cases a marked increase in enzyme stability. Many studies about enzyme immobilization by covalent and non-covalent bonding to solid supports and encapsulation have been developed [6,7,8,9] where most of the catalyst mass is inert support. A sound alternative is the use if carrier-free systems in which case the enzyme protein constitutes its own support so that most of the catalyst mass is active and very high specific activities are attainable and no support (which sometimes is more expensive than the enzyme itself) is required [10]. Cross-linked enzyme aggregates (CLEAs) are produced by cross-linking of enzyme protein aggregates produced by conventional non-denaturing protein precipitation techniques, being an attractive option to conventional immobilization in solid inert carriers. CLEAs share the good properties of carrier-free systems (simplicity and high specific activity) without the drawback of high production cost, since no purified enzyme is required as starting material, as opposite to cross-liked enzyme crystals [11]. Several conventional techniques used for protein purification are applicable, obtaining high recovery yields of enzyme activity. Immobilization by aggregation and crosslinking appears as a very promising strategy for enzyme co-immobilization [12,13] and several enzymes applied in sequential biocatalytic processes have been co-immobilized in the format of CLEAs offering the potential advantages of the proximity of the enzymes which will result in increased productivity. In addition, the use of co-immobilized enzymes will reduce the number of unit operations and reactor volume, and will reduce waste generation [14,15,16,17,18,19]. Catalysts produced by this strategy are called combi-CLEAs. Ahumada et al. [20] reported the preparation of an ARA-βG combi CLEAs obtaining good immobilization yields of expressed activity with both enzymes, with a marked increase in stability at simulated winemaking conditions.

The purpose of this work was the evaluation of the potential of the synthesized combi-CLEAs, obtained from the soluble commercial enzyme AR2000, as catalysts for the release of the compounds responsible for the aroma of Muscat wine, comparing its performance with the performance obtained with the soluble commercial enzyme AR2000 and obtained without enzyme addition (control).

## 2. Results and Discussion

### 2.1. Preparation and Characterization of Combi-CLEAs

Combi-CLEAs were prepared as described before by Ahumada et al. [20], obtaining an immobilization yield of expressed activity (IY) of 71.4% and 55.7% for βG and ARA respectively. Specific activities of combi-CLEAs were 14.98 and 11.97 IU/g catalyst for βG and ARA respectively. Even though it is desirable to obtain high IY for both enzymes, IY for βG is more relevant since βG will act not only on the product of reaction of ARA, but also on the products of RAM and API reactions as well (see Scheme 1); in fact, these activities are also present in the enzyme preparation and will act on wine contributing to a higher concentration of substrate to be acted upon by βG.

### 2.2. Stability of Combi-CLEAs

**Figure 1:**
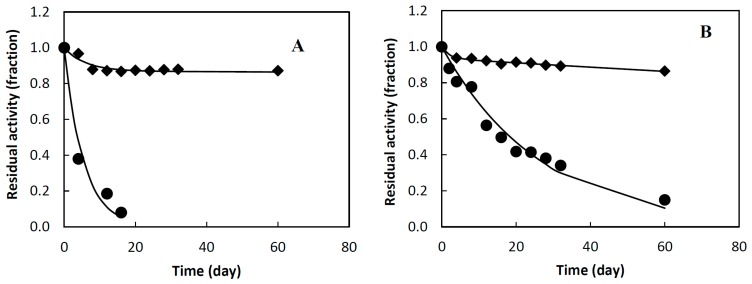
Stability of the catalyst in the presence of Muscat wine at 16 °C. (**A**) soluble (●) and immobilized (◆) βG; (**B**) soluble (●) and immobilized (◆) ARA. Lines represent the inactivation models.

In order to assess the stability of the biocatalysts in wine, the combi-CLEAs and the soluble enzyme preparation were incubated in Muscat wine. In the case of combi-CLEAs no enzyme leakage was observed during the whole incubation period in wine. As shown in Figure 1, co-immobilized enzymes were much more stable than their soluble counterparts.

Values of inactivation parameters are presented in Table 1. Inactivation models were used for estimating biocatalyst half-life (t_1/2_), defined as the time at which residual enzyme activity is half of its initial value, and stabilization factor (SF), defined as the t_1/2_ ratio of the immobilized and soluble enzymes. Very high values of SF were obtained for both enzymes, so that t_1/2_ were determined by extrapolation of the experimental data according to the corresponding models of inactivation. Previously, Ahumada et al. [20] showed that βG and ARA immobilized in combi-CLEAs were much more stable than their soluble counterparts when incubated at simulated winemaking conditions: pH 3.5, 10% (*v/v*) ethanol and 25 °C. Under such conditions SF were 33.8 and 8.9 for βG and ARA respectively. Values of SF obtained in this work are much higher than those, which can be attributed, at least in part, to the lower incubation temperature but also to the protective effect of Muscat wine, which is certainly an important asset from a technological perspective. González-Pombo et al. [21] reported the co-immobilization of βG, ARA, and RAM from *Aspergillus niger* in Eupergit C, obtaining also significant stabilization of βG and ARA with no decrease in activity after 70 days of incubation at simulated winemaking conditions (12% ethanol, 3.5 g/L tartaric acid, 2.5 g/L malic acid and 60 mg/L sodium metabisulfite). These results are certainly promising but not comparable with this work because of the different conditions used.

### 2.3. Volatile Compounds in Muscat Wines

The aroma composition (terpenes, alcohols and esters) of Muscat wine obtained from the treatments with soluble enzyme and combi-CLEAs, and without enzymatic treatment (control) is shown in Table 2. Total terpenes release was significantly higher in enzyme- treated wines than in the control.

Terpenes are key compounds in determining the flavor and varietal character of *Vitis vinifera* cultivars with an important contribution to the floral and citrus characters of wines [22,23]. The analysis of variance (ANOVA) demonstrated that treating wine with glucosidase enzymes has a significant effect on the release of glycosides, showing an increase in total terpenes from 2526 µg/L in the control wine to 2900 and 2999 µg/L in wines treated with soluble enzymes and combi-CLEAs respectively. Even though difference in total terpenes between both enzymatic treatments was not significant, the analyses were done at a fixed time, so the evolution in time of the profile was not determined which is expected to favor combi-CLEAs over the soluble enzymes due to the higher operational stability of the former (Figure 1).

Table 2 shows that significantly higher concentrations of linalool, nerol and geraniol were obtained in enzyme-treated wines than in the control and the highest values were obtained with combi-CLEAs. These three terpenes are the most important in determining Muscat wine typicity [24], and are used as an index for the classification of varieties into aromatic or non-aromatic [25,26]. These terpenes, like the others present, are mostly in glycosylated form in grapes and are released during fermentation and aging of wine. All of them were present in our wines, but only linalool and geraniol concentrations were higher than their respective odor thresholds, contributing directly to the aroma, providing floral, citrus, lavender, citric and geranium aromas [27,28]. The wine treated with combi-CLEAs was the one presenting the highest concentration of these volatile compounds probably due to the higher stability of this catalyst during the process of aroma release.

Using combi-CLEAs, the concentrations of linalool, nerol and geraniol were 20%, 20% and 100% higher than in the control, respectively. These differences were clearly perceived by trained panelists, (data not shown). Results obtained with respect to linalool and geraniol were similar than those reported by González-Pombo et al. [21] and Romo-Sanchéz et al. [29] in Muscat wines when using co-immobilized glycosidases. In the case of α-terpineol, another important component in Muscat variety [30], the concentration obtained was 9% higher in treated wine with combi-CLEAs than in the control wine; Table 2 also shows increased concentrations of other terpenes like (*Z*)-linalooloxide (furanoid) and 2,6-dimethyl-1,7-octadiene-3,6-diol. On the other hand, concentrations of citronellol, (*E*)-linalooloxide (pyranoid), (*Z*)-linalooloxide (pyranoid), hotrienol and (*E*)-8-hydroxylinalool showed no significant differences among treatments. Moreover, these compounds had concentrations lower than their odor threshold not having a direct influence on wine aroma.

The three wines produced were also analyzed in their content of alcohols and esters. Among alcohols, benzyl alcohol was the most affected by the enzyme treatment, whether with the soluble or with the combi-CLEAs. However, the increase observed is not significant in terms of aroma considering the high olfactory perception threshold of alcohols. Romo-Sánchez et al. [29] also reported an increase in benzyl alcohol and other alcohols when wine was treated with glycosidases. This may be due to the fact that a small portion of these compounds coming from grape skins are found in glycosylated form [26,31] and may be hydrolyzed by the glycosidase enzymes. With respect to esters, the concentrations of some of them are higher than their olfactory perception threshold providing fruity aromas to wine [32]. Interestingly, only the wines treated with combi-CLEAs exhibited increased levels of hexyl acetate, ethyl hexanoate, ethyl octanoate and diethyl succinate. There is no background information on the effect of glycosidases on their content in wine, which is to be expected since the presence of these compounds is likely to be related to the use of enzymes in the maceration stage of winemaking [33].

### 2.4. Treatments Classification

**Figure 2:**
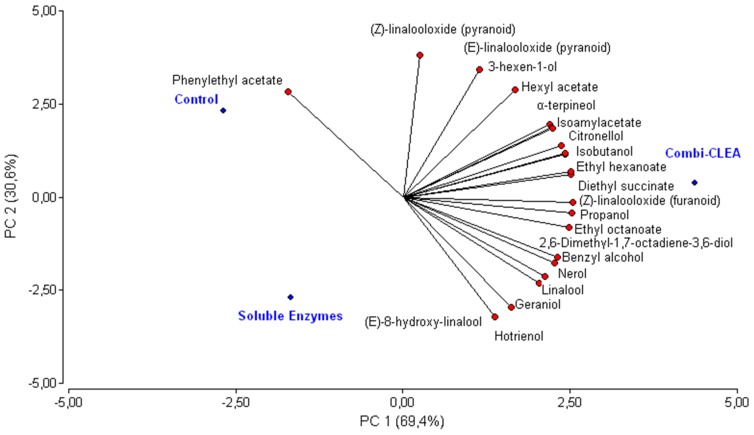
Principal components analysis (PCA) performed with volatile composition (µg/L) in Muscat wines from untreated, control, and treated wines with soluble enzymes and combi-CLEAs.

Principal components analysis (PCA) was performed for assessing whether the effects of the different enzymes allowed classifying the wines according to their volatile composition (Figure 2). Principal component 1 (PC 1) explained 69.4% of the variance and principal component 2 (PC2) explained 30.6%, representing 100% of all the variance. PC 1 was strongly correlated with citronellol, α-terpinol, (*Z*)-linalooloxide (furanoid), 2,6-dimethyl-1,7-octadiene-3,6-diol, propanol, isobutanol, isoamyl acetate, ethyl hexanoate, hexyl acetate, ethyl octanoate, diethyl succinate and benzyl alcohol, while PC 2 was strongly correlated to (*E*)-linalool oxide (pyranoid), and (*Z*)-linalool oxide (pyranoid).

The wine produced with combi-CLEAs was clearly distinguished from the other wines, since the PC 1 function, being the one separating them, had more height of the variance in the statistical analysis. Treatment with combi-CLEAs was correlated to the higher content of volatile compounds such as all terpenes, alcohols and esters; only phenylethyl acetate concentration was higher in the control wine. Therefore, this result indicates that the release of glycosylated aromatic compounds was higher when treated with combi-CLEAs. Also, the wine treated with the soluble enzymes could be distinguished from the control wine, but mostly by the PC2 that represents only 30.6% of the variance, so the differences between them were low; this sustains that the release of glycosylated compounds was the highest with combi-CLEAs, significantly lower with the soluble enzymes, and still lower in the control wine.

The three most important compounds for Muscat wine aroma typicity, namely linalool, geraniol and nerol, showed a negative correlation with phenylethyl acetate, a compound more related to the control wine. The control wine presented a lower concentration of these three aromatic compounds, being the most probable cause of having a low floral and fruity peculiar aroma. Enological parameters of the three types of wine produced are presented in Table 3 showing no significant differences among them.

## 3. Materials and Methods

### 3.1. Materials

The commercial enzyme preparation Rapidase^®^ AR 2000 was from DSM (Delft, The Netherlands) and was characterized in terms of total protein content and main glycosidase activities. Protein content, according to Bradford [36], was 15.24 ± 0.35 mg protein/g, βG specific activity was 8.07 ± 0.39 IU/g, and ARA specific activity was 8.26 ± 0.28 IU/g. Bovine serum albumin (BSA) was from Loba Chemie (Mumbai, India). *p*-Nitrophenyl-α-L-arabinofuranoside (pNPA), *p*-nitrophenyl-β-d-gluco-pyranoside (pNPG), and glutaraldehyde grade II (25% *v/v*) were from Sigma-Aldrich (St. Louis, MO, USA). Ammonium sulfate was from Merck (Darmstadt, Germany). All other reagents were of the highest available purity and used as purchased.

### 3.2. Hydrolytic Activity

The enzymatic activities of soluble and immobilized ARA and βG were determined using pNPA and pNPG as substrates, respectively, measuring the *p-*nitrophenol (pNP) release spectrophotometrically at 405 nm using a magnetically-stirred temperature-controlled cell [20]. To initiate the reaction, 200 μL of the enzymatic solution or suspension were added to 1.8 mL of a solution containing 0.42 mM of pNPA or 0.61 mM of pNPG for measuring ARA or βG activity, respectively. One international unit of ARA and βG activity (IU) was defined as the amount of enzyme producing 1 μmole of pNP per minute at 40 °C and pH 7 (100 mM phosphate buffer) from the corresponding *p*-nitrophenyl derivative. The molar extinction coefficient of pNP was 8.462 mM^−1^.

### 3.3. Preparation of Combi-CLEAs

**Scheme 2:**
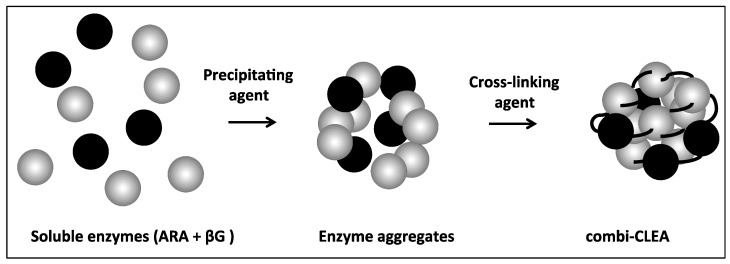
Preparation of combi-CLEAs.

Combi-CLEAs were prepared as reported in detail by Ahumada et al. [20]. A solution was prepared containing 0.13 g of commercial enzyme preparation per mL of 100 mM phosphate buffer pH 7. Bovine serum albumin was then added at an enzyme protein/albumin mass ratio of 3. Afterwards, 40 mL of ammonium sulfate (570 mg/mL) were added under stirring at 300 rpm, temperature being kept below 4 °C. After adding the precipitating agent, the suspension was maintained under stirring at 300 rpm for 30 min and then centrifuged at 10,000× *g* for 20 min at 3 °C. A volume of 25 mL of the supernatant was discarded and the precipitate was suspended in the remaining volume. Enzyme protein was then crosslinked by slowly adding 4 mL of 25% (*v/v*) glutaraldehyde at 4 °C under stirring at 300 rpm; after adding the crosslinking agent the suspension was maintained under stirring for 1 h at the same conditions. The suspension containing the combi-CLEAs was centrifuged at 10,000× *g* for 20 min at 3 °C, discarding the supernatant. The combi-CLEAs were suspended in 100 mM phosphate buffer pH 7 and again centrifuged at 10,000× *g* for 20 min at 3 °C. This washing procedure was repeated three times in order to remove the unreacted glutaraldehyde. The biocatalysts were suspended in 100 mM phosphate buffer pH 7 and stored at 4 °C (Scheme 2).

Two parameters were determined for each of the immobilized enzymes in the combi-CLEAs: immobilization yield of expressed activity (IY) and specific activity of the catalyst (IU/g catalyst). IY was defined as the percentage ratio of the units of activity expressed in the combi-CLEAs (A_I_) and the units of activity subjected to immobilization (A_C_), as shown in Equation (1):

(1) $$IY=\frac{A_{I}}{A_{C}}\cdot 100$$

### 3.4. Stability of Combi-CLEAs

The stability of the enzymes in the combi-CLEAs (0.07 mg biocatalyst/mL) and the soluble enzymes (0.05 mg commercial enzyme/mL) was evaluated in Muscat wine at 16 °C, determining residual activities of ARA and βG at regular time intervals. The experiments were carried out in triplicate and standard error was always below 5%.

Inactivation kinetics was modeled according to different inactivation mechanisms as proposed by Henley and Sadana [37,38]; inactivation parameters were determined from the best-fit model of the experimental data.

The mechanism based on two-stage series inactivation mechanism without residual activity was selected for better describing the inactivation kinetics of the enzymes in combi-CLEA. This mechanism is represented by the following scheme:

$$E\overset{k_{1}}{\to }E1\overset{k_{2}}{\to }E2$$

where *k*_1_ and *k*_2_ are first-order transition rate constants, and *E*, *E*_1_ and *E*_2_ are the corresponding enzyme species of progressively lower specific activity, being the last one completely inactive. The mathematical model representing this mechanism is:

(2) $$a=\left(1+\alpha \cdot \left(\frac{k_{1}}{k_{2}-k_{1}}\right)\right)\cdot exp^{\left(-k_{1}\cdot t\right)}-\left(\alpha \cdot \left(\frac{k_{1}}{k_{2}-k_{1}}\right)\right)\cdot exp^{\left(-k_{2}\cdot t\right)}$$

where a represents the residual activity at time *t* and α is the ratio of specific activity of enzyme species *E*_1_ with respect to that of the native enzyme species *E*.

The mechanism better describing inactivation in the case of soluble enzymes was one-stage first-order inactivation without residual activity. In this case α = 0, so the mathematical model is simply:

(3) $$a=exp^{\left(-k_{1}\cdot t\right)}$$

t_1/2_ was determined by interpolation or extrapolation from the respective models described by Equation (2) or (3). SF was the parameter used for a quantitative comparison of the stability of the biocatalysts at winemaking conditions. The coefficient of determination, R^2^, was determined in each case.

### 3.5. Winemaking

White Muscat grapes were harvested on 18 March 2016 at the ripening moment when the Baume degrees/titratable acidity ratio was 3.28. The grapes were destemmed and crushed to obtain the must. Seven grams of potassium metabisulfite per 100 kg of grapes were added. The must was introduced into a closed pneumatic press, where it remained in contact with the grape skins for 6 h at 6 °C. During this maceration, 2 g of Lafazym Press enzyme preparation (Laffort, Santiago, Chile) per 100 kg of vintage were added for increasing the extraction of precursors. Grapes were pressed with 55% yield. The must, without skin contact, was put in a 400 L stainless steel tank. A static racking at 10 °C for about 24 h was performed up to average values of 150–200 NTU (nephelometric turbidity units). *Saccharomyces cerevisiae* strain BO213 was inoculated at a dose of 20 g/hL according to the recommendation of the enzyme supplier. The alcoholic fermentation temperature was maintained around 16 °C, and the density of the medium was measured daily with a densimeter. The alcoholic fermentation was completed when the reducing sugars were below 2.5 g/L. At the end of it, the lees were removed and the free SO_2_ concentration was adjusted to 25–35 mg/L.

### 3.6. Enzymatic Treatment of Wine

The wine was divided in three stainless steel tank reactors for each treatment. The first reactor corresponded to the one where the treatment with soluble enzyme (AR 2000) was done using 3.5 g of enzyme preparation per 100 L of Muscat wine. The second reactor corresponded to the one where the treatment with combi-CLEAs was done at a dose of 5 g of catalyst per 100 L of Muscat wine. The third reactor corresponded to the one where the control without enzyme addition was done. All reactors were incubated in a temperature-controlled chamber at 16 °C during seven weeks. Reactors were shaken once per week for favoring the contact of the enzyme with the wine. After this time, the wine was clarified with 3g/hL of Vegecoll protein (Laffort) and samples from the three reactors were taken for analysis.

### 3.7. Oenological Parameter Analysis

Titratable acidity (g/L tartaric acid), pH, volatile acidity (g/L acetic acid) and alcohol degree were determined according to the methodology established by the International Organisation of Vine and Wine [39].

### 3.8. Analysis of Volatile Compounds by GC-MS

Volatile compounds were extracted following a previously reported methodology [40]. Prepacked cartridges (total volume 3 mL) filled with 200 mg of LiChrolut EN resin (Merck) were used for this extraction. Before passing the wine through the cartridge, 500 µL of internal standard (2-octanol) were added in absolute ethanol solution (Merck). The separation, identification and quantification of volatile compounds from the wine were carried out using an Agilent 7890A gas chromatograph, coupled with a 5975C mass spectrometer (Agilent Technologies Inc., Santa Clara, CA, USA). The unit was equipped with a fused silica capillary column (length 30 m × 0.25 mm id., and 0.5 μm phase thickness, DB-Wax, J & W Scientific, Agilent). The injector temperature was set at 250 °C. The carrier was helium applied at a flow rate of 1 mL/min. The temperature of the injector was 250 °C and 2 μL were injected. The oven temperature was initially held at 40 °C for 5 min, then increased linearly at a rate of 2 °C/min up to 130 °C and held at that temperature for 5 min; after that, temperature was again increased at a rate of 2 °C/min up to 180 °C and held at that temperature for 2 min; finally, the temperature was increased linearly at a rate of 4 °C/min for 230 min. The transfer line was 250 °C. The analysis was carried out with injection in splitless mode. Ionisation was carried out by electron impact at 70 eV. The operating method was a scan mode at *m*/*z* between 30 and 300, except for terpenes and C13-norisoprenoids, where the sim mode was used. Identification was carried out using the NIST library. When standards were available, the quantification was based on seven-point calibration curves of the respective standards (Sigma-Aldrich, Steinheim, Germany) (*R*^2^ > 0.93) in a 12% (*v/v*) ethanol solution at pH 3.6; otherwise, semi-quantitative analyses were carried out using the calibration curves of the most similar compound.

### 3.9. Statistical Analysis

Statistical analysis was carried out using SPSS Version 21.0 statistical package for Windows (SPSS Inc., Chicago, IL, USA). Volatile compounds data were processed using variance analysis (ANOVA). Differences between means were compared using the least significant difference (LSD) test at 0.05 probability level. PCA was performed using InfoStat (www.infostat.com.ar).

## 4. Conclusions

Combi-CLEAs of βG and ARA were prepared with high immobilization yields for both enzymes. The enzymes in combi-CLEAs were highly stable under conditions of winemaking with SF values of 1460 and 843 for βG and ARA respectively, which are much higher than reported in the literature for these enzymes. This result is at least in part due to an adequate strategy of immobilization but also due to the low temperature of operation and the protective effect of Muscat wine components, which is certainly an important asset from a technological perspective.

Highest release of total terpenes in wine was obtained with combi-CLEAs, their concentration being 18% higher than in the control wine. Also the concentration of the most important terpenes in terms of aroma and Muscat typicity, namely linalool, nerol and geraniol, were the highest, being 20%, 20% and 100% higher than in the control wine respectively.

Co-immobilized enzymes were highly stable at winemaking conditions, so it is possible to consider their reutilization making it technologically attractive by reducing the impact of the enzyme cost on winemaking cost.

To the best of our knowledge, this is the first report in which combi-CLEAs have been used for aroma release in wine and enzymes stability being determined under winemaking conditions including a thorough characterization of the aroma profiles obtained.

## Figures and Tables

**Table 1:** Inactivation parameters of soluble and immobilized βG and ARA in Muscat wine at 16 °C. t_1/2_: half-life; SF: stability factor, as defined in the text; t_1/2_ for combi-CLEAs were determined by extrapolation of the experimental data according to the corresponding models of inactivation.

Biocatalyts	Parameters					
	a	k_1_ (d^−1^)	k_2_ (d^−1^)	R^2^	t_1/2_ (d)	SF
βG soluble		0.18		0.94	3.8	
βG in combi-CLEA	0.87	0.17	0.0001	0.85	5537	1460
ARA soluble		0.04		0.95	18.4	
ARA in combi-CLEA	0.93	0.48	0.00134	0.97	15510	843

**Table 2:** Volatile compounds content in Muscat control wines and Muscat wines treated with soluble enzymes and combi-CLEAs.

Compounds	Odor Threshold (Reference)	Control	Soluble Enzymes	Combi-CLEA
Terpenes µg/L				
Linalool	25 [27]	415.98 ± 0.98 a	469.62 ± 32.14 ab	495.83 ± 43.26 b
Citronellol	100 [27]	67.23 ± 1.40 ab	65.75 ± 2.61 b	71.74 ± 3.11 b
Nerol	400 [27]	270.59 ± 8.81 a	300.64 ± 2.36 b	322.81 ± 10.40 c
Geraniol	30 [28]	265.58 ± 4.30 a	499.32 ± 47.24 b	587.58 ± 11.52 c
α-Terpineol	250 [27]	177.89 ± 4.22 b	165.92 ± 2.80 a	194.71 ± 4.72 c
(*Z*)-Linalooloxide (furanoid)	6000 [34]	18.33 ± 0.34 a	18.27 ± 0.1.29 a	20.65 ± 0.14 b
Hotrienol	110 [30]	19.40 ± 1.03 a	26.93 ± 4.49 b	27.20 ± 0.21 b
(*E*)-Linalooloxide (pyranoid)	3000 [34]	144.99 ± 9.00 b	126.6 ± 9.22 a	146.61 ± 0.19 b
(*Z*)-Linalooloxide (pyranoid)	3000 [34]	61.26 ± 0.03 a	55.15 ± 5.43 a	59.45 ± 0.06 a
2.6-Dimethyl-1.7-octadiene-3.6-diol		143.25 ± 2.83 a	151.22 ± 16.05 ab	166.41 ± 3.15 b
(*E*)-8-Hydroxylinalool (trans-2.6-dimethyl-2.7-octadien-1.6-diol)		27.05 ± 2.12 a	124.17 ± 5.94 b	115.21 ± 5.65 b
**Total**		2526.62 a	2900.75 b	2999.01 b
Alcohols µg/L				
Propanol	750,000 [27]	22,141.24 ± 2293.86 a	22,598.11 ± 1577.10 a	24,665.39 ± 952.10 a
Isobutanol	40,000 [28]	33,865.40 ± 228.71 a	33,506.26 ± 3617.49 a	35,440.50 ± 3970.30 a
Benzyl alcohol	200,000 [35]	155.98 ± 6.42 a	219.44 ± 0.93 b	274.53 ± 48.76 b
*cis*-3-Hexen-1-ol	400 [27]	154.48 ± 8.58 ab	135.64 ± 3.16 a	163.75 ± 18.55 b
**Total**		56317.09 a	56459.46 a	60544.17 a
Esters µg/L				
Isoamylacetate	30 [35]	7725.37 ± 264.72 a	7505.14 ± 45.35 a	8090.17 ± 563.83 a
Hexyl acetate	670 [35]	380.17 ± 17.74 a	364.70 ± 14.25 a	406.99 ± 3.86 b
Phenylethyl acetate	250 [27]	1722.54 ± 1.97 b	1579.22 ± 46.55 a	1564.80 ± 50.11 a
Ethyl hexanoate	14 [32]	960.09 ± 15.49 a	923.50 ± 46.01 a	1117.61 ± 14.39 b
Ethyl octanoate	5 [32]	1589.86 ± 45.16 a	1689.92 ± 99.26 a	1985.06 ± 51.83 b
Diethyl succinate	500,000 [32]	1019.75 ± 84.30 a	1005.84 ± 75.63 a	1254.76 ± 24.37 b
**Total**		13,397.78 ab	13,068.32 a	14,419.4 b

All parameters are given with their standard deviation (*n* = 3). Different letters in the same row indicate significant differences (*p* ≤ 0.05) between treatments according to the ANOVA test.

**Table 3:** Oenological parameters of Muscat wine.

Treatment	pH	Total Acidity (g/L) ^a^	Volatile Acidity (g /L) ^b^	Alcohol (*v/v* %)
Control	3.97	3.67	0.32	13.0
Soluble enzymes	3.99	3.53	0.38	13.0
Combi-CLEA	3.98	3.63	0.34	13.1

^a^ As g/L tartaric acid; ^b^ As g/L acetic acid.
